# Transmembrane capability of DNA origami sheet enhanced by 3D configurational changes

**DOI:** 10.1016/j.isci.2023.106208

**Published:** 2023-02-15

**Authors:** Fengyu Liu, Xiaoming Liu, Wendi Gao, Libo Zhao, Qiang Huang, Tatsuo Arai

**Affiliations:** 1 Key Laboratory of Biomimetic Robots and Systems, Ministry of Education, State Key Laboratory of Intelligent Control and Decision of Complex System, Beijing Advanced Innovation Center for Intelligent Robots and Systems, and School of Mechatronical Engineering, Beijing Institute of Technology, Beijing 100081, China; 2 State Key Laboratory for Manufacturing Systems Engineering, International Joint Laboratory for Micro/Nano Manufacturing and Measurement Technologies, and School of Mechanical Engineering, Xi’an Jiaotong University, Xi’an 710049, China; 3 Center for Neuroscience and Biomedical Engineering, The University of Electro-Communications, Tokyo 182-8585, Japan

**Keywords:** Materials science, Materials chemistry, Biomaterials, Materials characterization

## Abstract

DNA origami-engineered nanostructures are widely used in biomedical applications involving transmembrane delivery. Here, we propose a method to enhance the transmembrane capability of DNA origami sheets by changing their configuration from two-dimensional to three-dimensional. Three DNA nanostructures are designed and constructed, including the two-dimensional rectangular DNA origami sheet, the DNA tube, and the DNA tetrahedron. The latter two are the variants of the DNA origami sheet with three-dimensional morphologies achieved through one-step folding and multi-step parallel folding separately. The design feasibility and structural stability of three DNA nanostructures are confirmed by molecular dynamics simulations. The fluorescence signals of the brain tumor models demonstrate that the tubular and the tetrahedral configurational changes could dramatically increase the penetration efficiency of the original DNA origami sheet by about three and five times, respectively. Our findings provide constructive insights for further rational designs of DNA nanostructures for transmembrane delivery.

## Highlights

• DNA origami sheet can be converted into DNA tube and DNA tetrahedron, respectively
• The 3D configuration of DNA tube and DNA tetrahedron contributes to their stability
• DNA tetrahedron penetrates more efficiently than DNA tube and DNA origami sheet

## Graphical abstract

**Figure:**
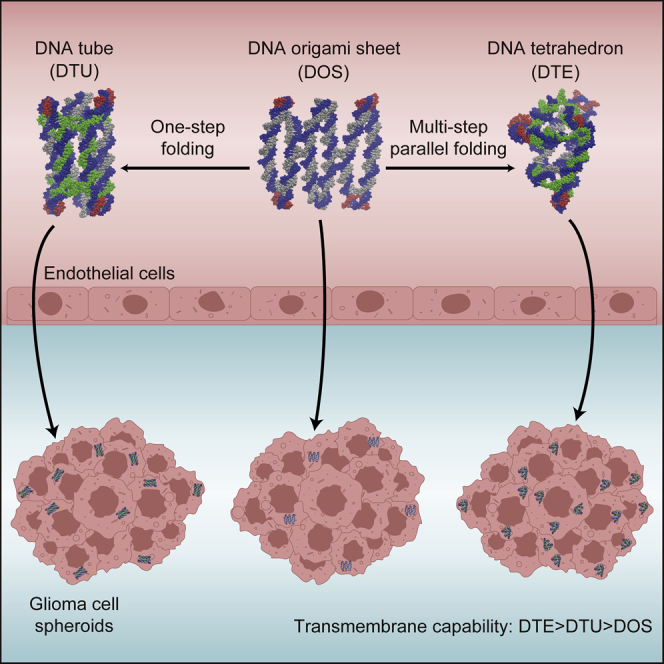

## Introduction

Due to the strong biocompatibility, easy assembly, and high fidelity,^1^^,^^2^^,^^3^ DNA origami has developed into a revolutionary technology for biomedical applications, mechanical motions, and secure communications.^4^^,^^5^^,^^6^^,^^7^ DNA origami-engineered nanostructures with different sizes and shapes are widely used in transmembrane delivery.^8^^,^^9^^,^^10^ Enhancing the transmembrane capability of the DNA origami-based nanostructures is crucial for delivering cargo to target cells,^11^^,^^12^^,^^13^^,^^14^ especially for crossing the blood–brain barrier and permeating brain tumors.^15^^,^^16^^,^^17^ Planar DNA origami sheets (DOS) have been frequently used as nanocarriers for transmembrane delivery since they were first proposed by Rothemund,^4^ and their transmembrane capabilities could be improved by employing external materials such as transferrin,^18^ virus capsid proteins,^19^ and cationic amino acids.^20^ However, binding additional materials to DOS usually requires functional cross-linkers and long waits,^18^^,^^19^ and the accidental separation of bound materials^18^^,^^19^^,^^20^ might affect the delivery efficiency. Hence, an efficient strategy to enhance the transmembrane capability of DOS for delivery crossing the blood–brain barrier is urgently required.

Recently, some studies have demonstrated that the size and geometry of DNA origami nanostructures could influence their transmembrane capability.^10^^,^^11^^,^^12^^,^^13^^,^^14^ Peng and co-authors noted that tetrahedral DNA frameworks interacted more distinctly with membrane receptors than triangular prisms and cubes.^13^ Moreover, Wang and collaborators declared that the larger DNA tubes were internalized into cancer cells significantly, but the smaller tubes and the DNA tetrahedrons were not.^12^ For planar DOS, converting them into three-dimensional (3D) variants may change their transmembrane capability. Interestingly, Li and colleagues bent the DOS into the tubular nanostructures to improve cellular uptake efficiency for targeted cancer therapy.^17^ In our previous work,^21^ we also found that folding the DOS into the tetrahedral nanostructures can significantly enhance the transmembrane efficiency to further recognize and internalize into the circulating tumor cells. However, there are no systematic studies yet to elucidate how the 3D configurational changes of DOS affect their penetration capability.

**Figure 1:**
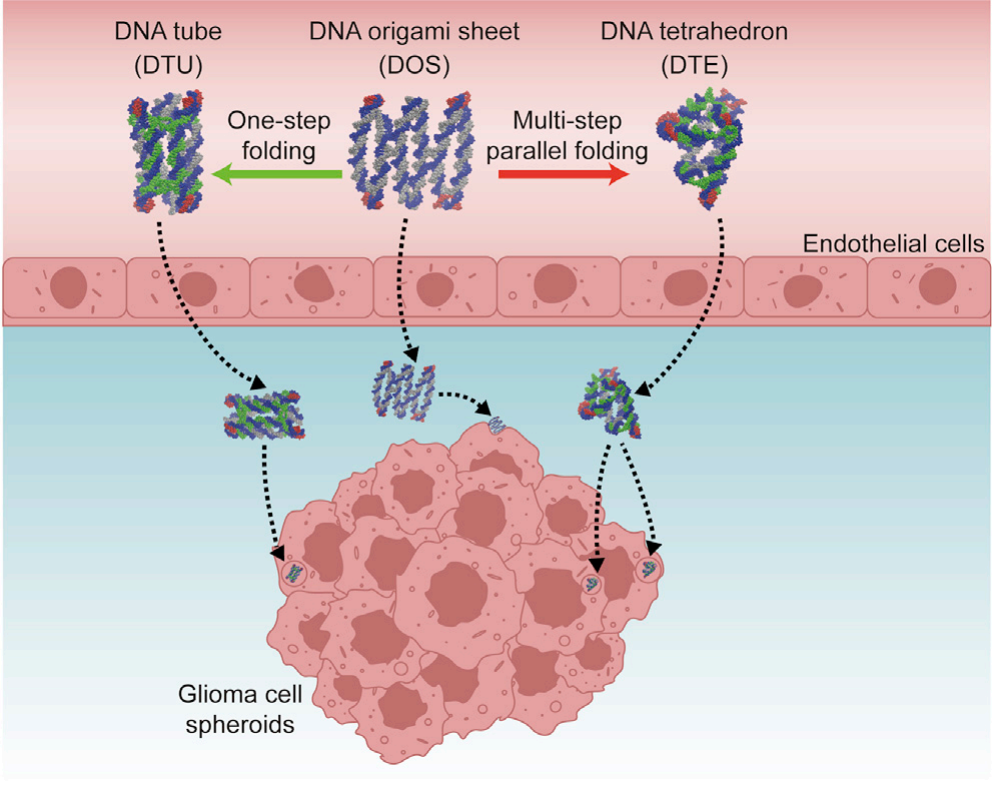
Transmembrane schematics of different DNA nanostructures in an *in vitro* 3D brain tumor model Three DNA nanostructures presented different permeability when they crossed bEnd.3 endothelial cell monolayers and subsequently infiltrated SF767 cell spheroids. One-step folding and multi-step parallel folding were adopted to convert DOS to DTU and DTE, respectively. Concrete folding strategies are shown in Figure S1.

To fill up the gap that no one has used tiny DNA nanostructures with diverse configurations to investigate their penetration differences in the presence of the blood–brain barrier, we herein design and construct three DNA nanostructures around 15 nm, including the two-dimensional (2D) rectangular DOS, the DNA tube (DTU), and the DNA tetrahedron (DTE). The latter two are the variants of the DOS with 3D morphologies achieved through one-step folding^17^ and multi-steps parallel folding^21^ separately. We investigate the transmembrane efficiency between DOS and its two 3D variants during permeating glioma cell spheroids with blood–brain barrier protection *in vitro*. We examine the design feasibility of three DNA objects with the same molecular weight by the simulated relaxation trajectories. We also characterize the spatial conformation and structural stability of three DNA objects. Finally, we incubate diverse DNA nanostructures with the 3D brain tumor model *in vitro* (Figure 1) to explore their dynamic transmembrane capabilities as they traverse bEnd.3 endothelial cell monolayers and subsequently infiltrate SF767 cell spheroids.

## Results

We used the caDNAno^22^ to design all three DNA nanostructures, including DOS, DTU, and DTE. All pre-planned paths of scaffold strands and staple strands for constructing three different DNA origami nanostructures were carefully presented in Figure S1. Considering that previous critical studies^16^^,^^17^^,^^23^ indicated that nanoparticles around 15 nm can easily pass through the blood–brain barrier, we designed the size of planar DOS as 14 nm × 15 nm × 2 nm. To assemble tubular DTU, we adopted the one-step bending method introduced by Ding and co-authors.^17^ Figure S2 showed that rectangular DOS could be bent into the tubular configuration according to the green dotted folding axis. In addition, the tetrahedral DTE could be obtained utilizing the parallel folding strategy^21^ to fold the whole DOS object along the red dotted folding axes (Figure S2). Considering the significant stress generated by the above folding, we skillfully employed three nucleotides flexibility located in the center of all folding strands to enhance the successful bending rate of DTU and DTE (Figure S1).

**Figure 2:**
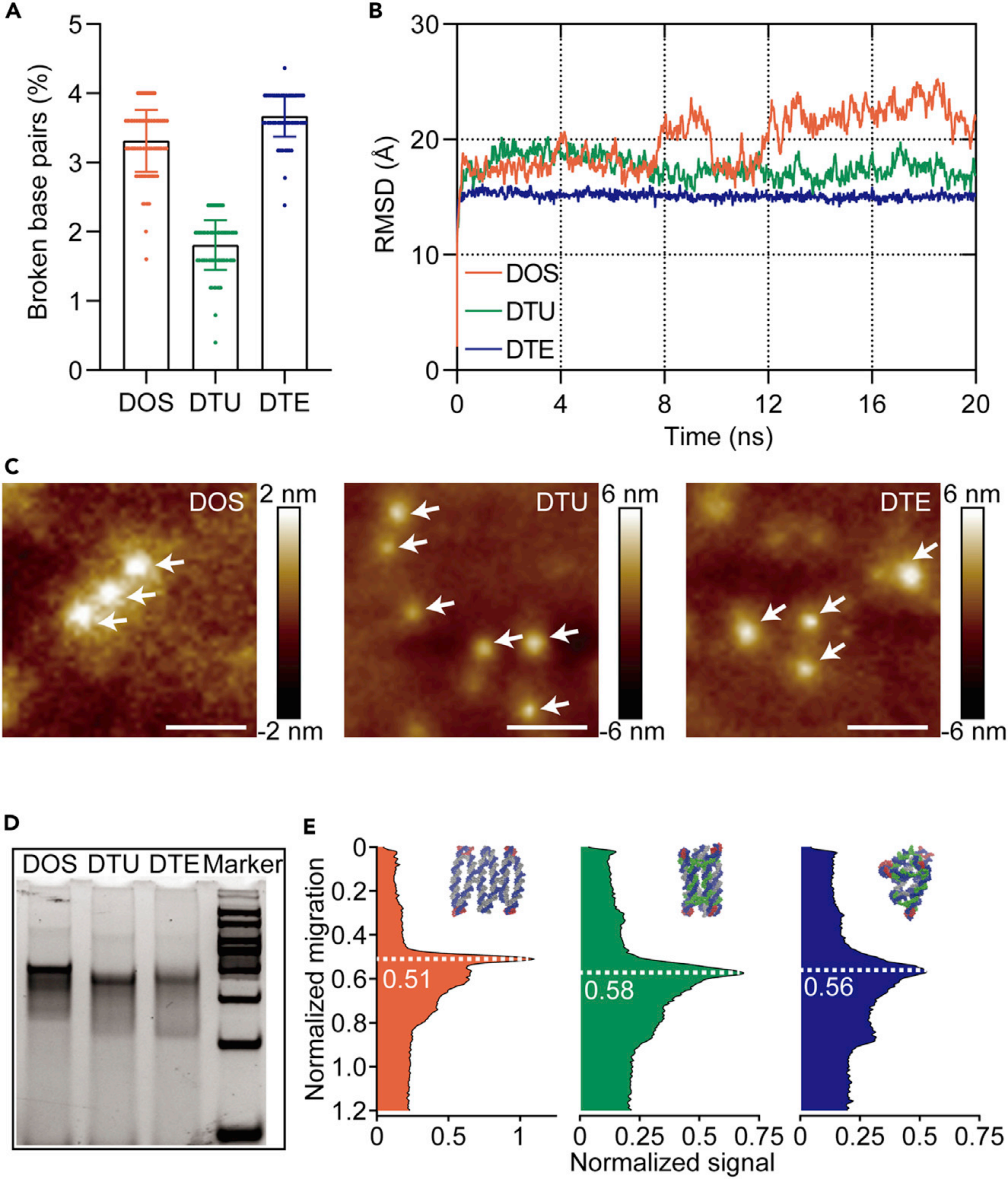
Simulation and characterization of various DNA nanostructures (A) The percent of base pairs breaks during the molecular dynamic (MD) simulations. When the distance between the N1 atom of the purine base and the N3 atom of the pyrimidine base is beyond 5 Å, the base pairs are considered broken. Data were presented as mean ± SD. (B) Root-mean-square deviation (RMSD) of the DNA atoms from their initial coordinates during the MD simulations. (C) Representative AFM images respectively showed the morphology of DOS, DTU, and DTE: rectangle, tube, and tetrahedron. White arrows point to the intact DNA nanostructures. Scale bars: 50 nm. (D) Typical native 10% PAGE data showed migrant differences among the DOS, DTU, and DTE. Marker (top to bottom): 500, 400, 300, 250, 200, 150, 100, and 50 bp. (E) Relative to the complete migration, the peak signal of DOS, DTU, and DTE is separately presented at 0.51, 0.58, and 0.56.

To analyze the design feasibility of 3D DNA objects (DTU and DTE) and predict the configuration of DOS, DTU, and DTE, we performed the multiresolution simulation of three nanostructures using the mrdna framework.^24^ After three rounds of calculations and predictions, the detailed equilibrium shapes of DNA variants were introduced to generate the NAMD configuration files. Given the predicted accuracy and computational cost, we chose the elastic network of restraints-guided molecular dynamics simulation to further characterize the conformation of three DNA objects without any solvent parameters.^25^ We separately analyzed the 20 ns simulated trajectories of three different DNA objects (Videos S1, S2, and S3). The fraction of broken base pairs during the molecular dynamics runs was obtained (Data S1). Even though there were 10 broken base pairs on DTE, the average fraction remained less than 4% among the three designs (Figure 2A). Moreover, we presented the root-mean-square deviation (RMSD) data of the DNA atoms from their initial coordinates (Figure 2B). Interestingly, the overall RMSD tendency of DTE was relatively smooth, suggesting the obtained equilibrium shape was stable. In contrast, dramatic RMSD fluctuation was monitored in DOS design as it possesses more unconstrained DNA double-helix regions than DTU and DTE.

Video S1. The fluctuation trajectory of DOS was shown by molecular simulations, related to Figure 2

Video S2. The fluctuation trajectory of DTU was shown by molecular simulations, related to Figure 2

Video S3. The fluctuation trajectory of DTE was shown by molecular simulations, related to Figure 2

To confirm the simulation accuracy of DNA nanostructures, we synthesized and characterized DOS, DTU, and DTE, respectively. We used the multi-step cooling method to assemble the plane DOS by annealing the long scaffold strands (Table S1) and all staple strands (Table S2). According to the specific folding strategies (Figure S2), we employed folding strands (Table S2) designed for the precise fabrications of tubular architecture and tetrahedral geometry to construct DTU and DTE. Afterward, these synthesized DNA nanostructures were characterized by using the peak force tapping mode of atomic force microscopy (AFM). The average height of the single-layer DOS was about 2 nm (Figure 2C), which indicates that the planar structure of the DOS was correctly constructed. Except for DOS, the feature structures of DTU and DTE are difficult to distinguish because the resolution of the obtained AFM images is not good enough. Many studies^13^^,^^20^^,^^26^ have attempted to profile similar tiny DNA nanostructures using AFM. However, the reported AFM images are not clear enough to show more details of tiny DNA nanostructures (around 15 nm). Taking into account the structural collapse caused by the continuous tapping of the AFM probe, the average height of both DTU and DTE was close to 6 nm (Figure 2C), suggesting that we have successfully obtained the desired 3D configurations. Moreover, these results are in line with the representative simulated morphologies of three DNA objects (Videos S1, S2, and S3). In addition to the AFM characterization, the diverse configurations of the three DNA nanostructures can be identified by their different mobility during gel electrophoresis separation. Therefore, we studied the migration differences of multiple DNA nanostructures by the polyacrylamide gel electrophoresis (PAGE) (Figure 2D). Notably, the normalized migration plot indicated that the peak signal value of DOS occurred at 0.51, implying that the migration speed of the planar DOS was the slowest during the gel electrophoresis (Figure 2E and Data S1). In contrast, the 3D DTU was the fastest, as its peak signal value appeared at 0.58. Briefly, the accuracy of multiple DNA nanostructure simulations was validated by diverse structural morphology in AFM and different mobility in PAGE.

The tolerance of DNA nanostructures to nuclease digestion and low-pH degradation is a critical prerequisite for their sustainable use.^11^ So, we analyzed the structural stability of DNA nanostructures treated with fetal bovine serum (10%) or low-pH (pH = 5) solutions utilizing PAGE. After incubating at different intervals, all DNA samples were run in 10% polyacrylamide gels for 1 h. We adopted a post-staining method to visualize the sample signals in recovered polyacrylamide gels. The structural stability differences among DOS, DTU, and DTE were elaborated in the typical gel images (Figures S3A and S3C) and the time-dependent results (Figures S3B and S3D). We found that nucleases ultimately degraded more than 60.4% DOS on average, and nearly three-quarters of DTU survived the entire incubation (6 h). Significantly higher levels of structural degradation of DNA nanostructures were found in low-pH solutions compared to cell culture media containing 10% fetal bovine serum (FBS) (Figures S3B and S3D). Considering the previous studies,^21^^,^^27^^,^^28^ we deduce that compact 3D configurations of tube and tetrahedron could resist the nuclease invasion and low-pH affection stronger than the planar DOS.

**Figure 3:**
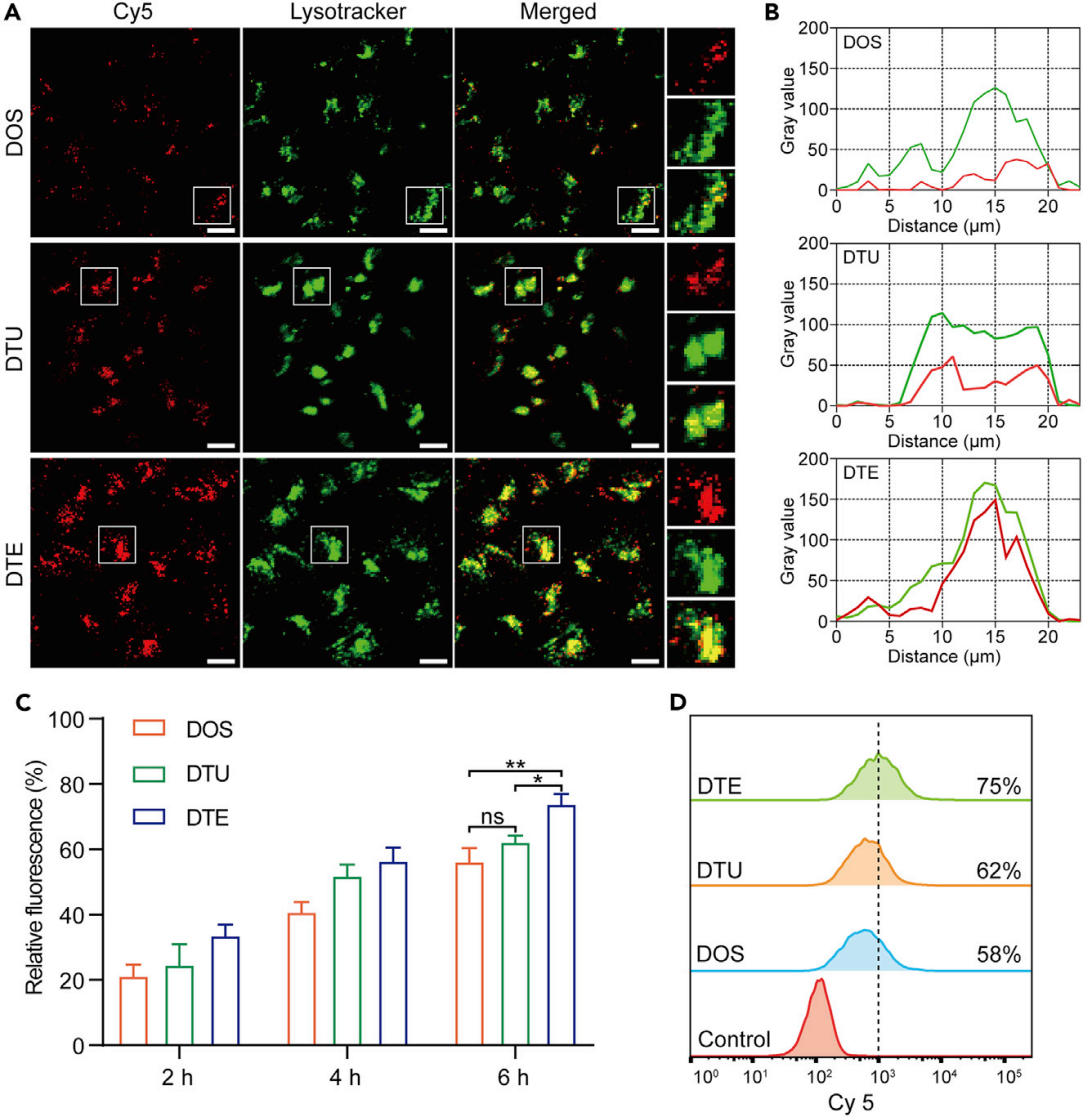
Cellular uptake study of different DNA nanostructures in HeLa cells (A) Confocal images showed that different amounts of DOS, DTU, and DTE were endocytosed into HeLa cells. All DNA nanostructures were labeled with Cy5. Lysotracker was employed to stain the lysosomes in HeLa cells. The enlarged images in the right column were chosen from corresponding white frame areas belonging to DOS, DTU, and DTE-treated groups. Scale bars: 20 μm. (B) Co-localization analysis of Cy5-modified DNA nanostructures and cellular lysosomes in the magnified regions of Figure 3A. The images of magnified regions were imported into ImageJ. The gray values of different images were obtained by using the Plot Profile tool of ImageJ. Red lines pointed to the Cy5 channel, and green lines referred to the lysotracker channel. (C) Flow cytometry analysis of cellular uptake efficiency of DNA nanostructures at different time intervals. Data were presented as mean ± SD. Comparisons were performed using the one-way ANOVA. ns, not significant. ∗p < 0.05. ∗∗p < 0.01. (D) Representative flow cytometry assay of HeLa cells treated with different DNA nanostructures for 6 h. The free Cy5 strands-treated group was considered the control group.

Several reports^29^^,^^30^^,^^31^ have suggested that strong structural stability is beneficial for the transmembrane delivery of DNA nanostructures. To inspect the above viewpoint in this work, we separately incubated DOS, DTU, and DTE with Hela cervical cancer cells to further measure their relative transmembrane capabilities. The successful hybridization of all three DNA nanostructures with Cy5 strands is essential to ensure the observation of DNA nanostructures under laser stimulation. During hybridization, high concentrations of Cy5 strands were introduced separately and then bound to the different Cy5 capture strands (Table S2) of three DNA nanostructures (Figure S1). Subsequently, Cy5-modified DNA nanostructures were filtered and recovered with the assistance of 30 kDa ultrafiltration devices. To validate the removal efficiency of excess Cy5 strands, we imaged gels containing diverse DNA nanostructures with the EtBr channel and the Cy5 channel, respectively. As shown in Figure S4B, there was no visible signal at the bottom of the whole gel, which indicated that the excess Cy5 strands were completely removed. Meanwhile, the merged image displayed that the co-localization of Cy5 dye and Cy5-modified DNA nanostructures was highly coincident (Figure S4C), suggesting that diverse DNA nanostructures can correctly incorporate with Cy5 strands. After the 6 h treatment of Cy5-labeled DNA variants, HeLa cells were stained with the lysotracker working solution for 30 min. Confocal microscopy results showed that more DTU and DTE were endocytosed into HeLa cells than DOS (Figure 3A). In addition, the enlarged images in the third row of Figure 3A displayed partial DTE escaped from cellular lysosomes. We analyzed these amplified graphs and depicted the co-localization charts between multiple DNA samples and cellular lysosomes to exhibit the cellular uptake efficiency of diverse DNA structures directly. The gray value distributions of all three groups were highly consistent between the red and green lines (Figure 3B and Data S1), revealing that red lines-pointed DNA samples were internalized into cellular lysosomes pointed by the green lines. In particular, the red line closest to the green line was presented in the DTE group, implying that more DTE samples were internalized into the cellular lysosomes than DTU and DOS. Given that the partial DNA samples could be digested in a cell culture environment (10% FBS), some Cy5 strands would disconnect from DNA samples and cause false-positive data. Therefore, we performed the control group of HeLa cells treated with free Cy5 strands to examine the cellular uptake efficiency of free Cy5 strands. The confocal images demonstrated that free Cy5 strands rarely entered the HeLa cells (Figure S5), verifying the reliability of the above confocal data.

Moreover, we investigated the transmembrane capabilities of three DNA nanostructures at different periods utilizing flow cytometry. Well-grown HeLa cells were cultivated with Cy5-modified DOS, DTU, and DTE for 2, 4, and 6 h at 37°C. The most pronounced fluorescence intensity was shown in DTE-treated HeLa cells regardless of incubation time (Figure 3C). More specifically, representative flow cytometry data showed that the percentages of Cy5 positivity in DOS, DTU, and DTE-treated HeLa cells were 58%, 62%, and 75%, respectively (Figure 3D), suggesting that maximal proportional DTE were endocytosed into HeLa cells during the whole-course cultivation. To further quantify the cellular uptake efficiency in three DNA nanostructures more precisely, we employed the qPCR method to analyze and calculate the relative DNA levels of three DNA nanostructures that were endocytosed into HeLa cells. Same Scaffold 2 (Table S1) belonging to all three DNA nanostructures was defined as the “target gene” and normalized to β-actin. Compared with DOS, nearly 1.5-fold DTU entered into HeLa cells (Figure S6). In addition, the relative DNA level of DTE was significantly higher than that of DTU. Collectively, the tetrahedral configuration has a higher transmembrane capability than the two other kinds, even though its structural stability was weaker than the tubular structures in physiological situations.

**Figure 4:**
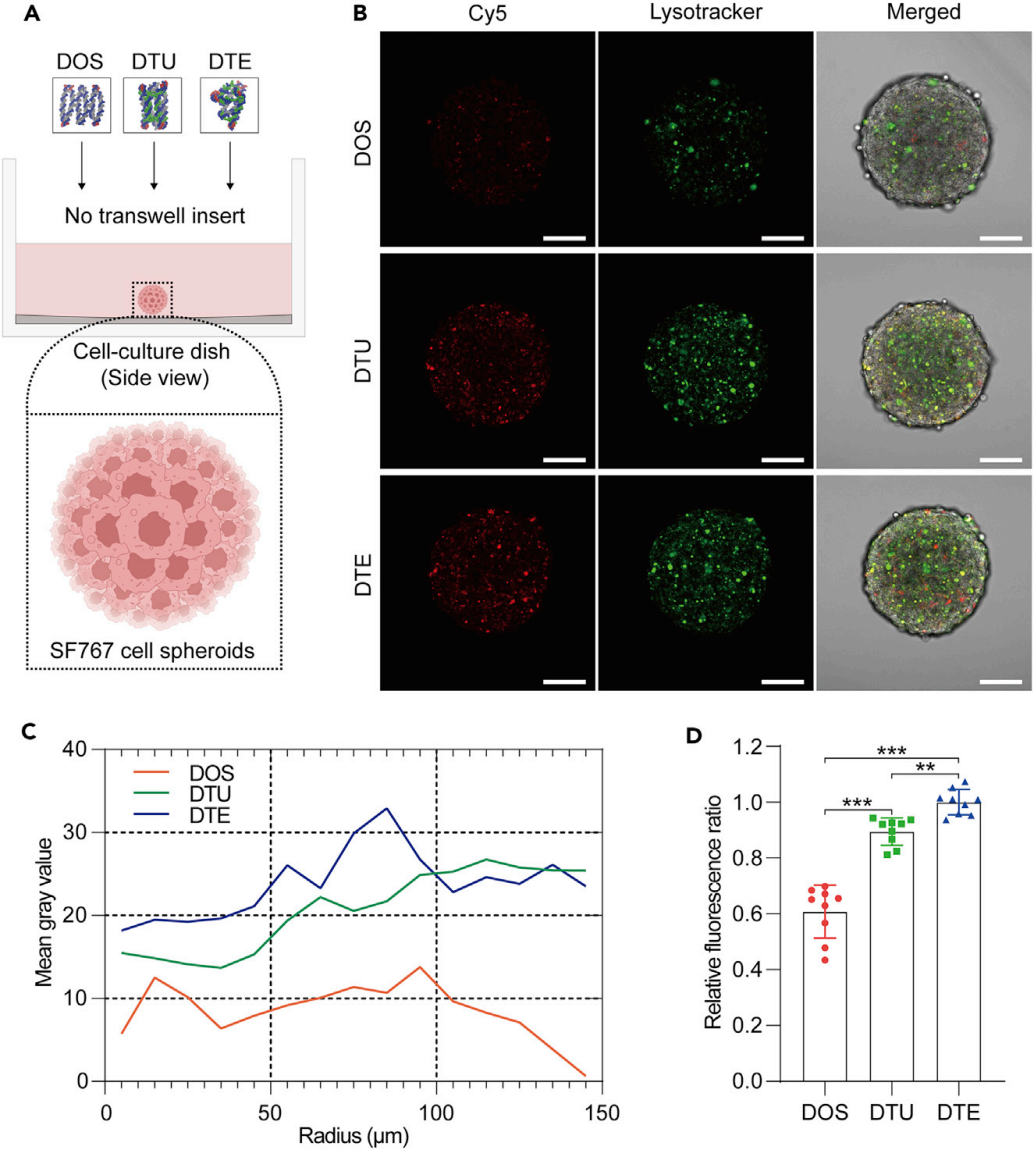
Comparison of the penetration efficiency of various DNA nanostructures into 3D tumor cell spheroids (A) The side view of the cell culture dish showed that the SF767 glioma cell spheroid is incubated with DOS, DTU, or DTE. The bottom illustration depicts the detailed construction of 3D tumor spheroids. (B) Cy5-modified DNA nanostructures penetrated the SF767 tumor spheroids. In all groups, the DNA nanostructures were treated for 6 h. Scale bars: 100 μm. (C) Radial fluorescence difference among multiple DNA nanostructures entered into the SF767 tumor spheroids. The radial fluorescence intensity of all cell spheroids was measured by a manual method. Briefly, 15 concentric circles with a radius gradually increased from 0 to 150 μm were manually selected on each cell spheroid using the ROI manager tool of ImageJ. The mean gray value between two adjacent rings was measured consecutively and then the line chart was generated. (D) Fluorescence statistic of the relative Cy5 intensity of DOS, DTU, and DTE in the corresponding SF767 tumor spheroids. All measured fluorescence results were normalized to the average value of the DTE group. The data shown represent mean ± SD from nine independent experiments. Comparisons were performed using one-way ANOVA. ∗∗p < 0.01, ∗∗∗p < 0.001.

To further verify that different spatial configurations also influenced the transmembrane capability of DNA samples used for brain cancer therapy, we explored the penetration efficiency of three DNA nanostructures when permeating the brain tumor models *in vitro*. Before permeating into brain tumors, various DNA nanostructures should cross the blood–brain barrier (BBB) monolayers successfully. Hence, we first constructed the BBB model *in vitro* prior to fabricating the brain tumor models. The appropriate bEnd.3 cerebrovascular endothelial cells were seeded into the gelatin-coated upper chamber of a transwell system (Figure S7A). Interestingly, fluorescence signal differences were observed among three DNA samples-treated bEnd.3 cells (Figure S7B), suggesting that various DNA nanostructures exhibited different penetration capabilities when directly confronting the BBB monolayers *in vitro*. In addition, various Cy5-labeled DNA samples were also directly incubated with SF767 glioma cell spheroids (Figure 4A). After 6 h of incubation, we determined the permeation capability of three DNA samples based on the relative fluorescence signals obtained from confocal microscopy scanning results. Stronger Cy5 fluorescence signals were observed in cell spheroids treated with DTU and DTE than with DOS (Figure 4B). We then performed a radial comparison of different Cy5 signals obtained from Figure 4B. The blue line is always higher than the orange and green lines from 0 to 100 μm (Figure 4C and Data S1), indicating that DTE is more efficient than DTU and DOS in infiltrating the center of the SF767 cell spheroids. Statistic histograms of relative fluorescence ratios of SF767 cell spheroids treated with different DNA nanostructures showed that the average percentages in the DOS and DTU groups were 60.8% and 89.4%, respectively, compared with the DTE group (Figure 4D).

**Figure 5:**
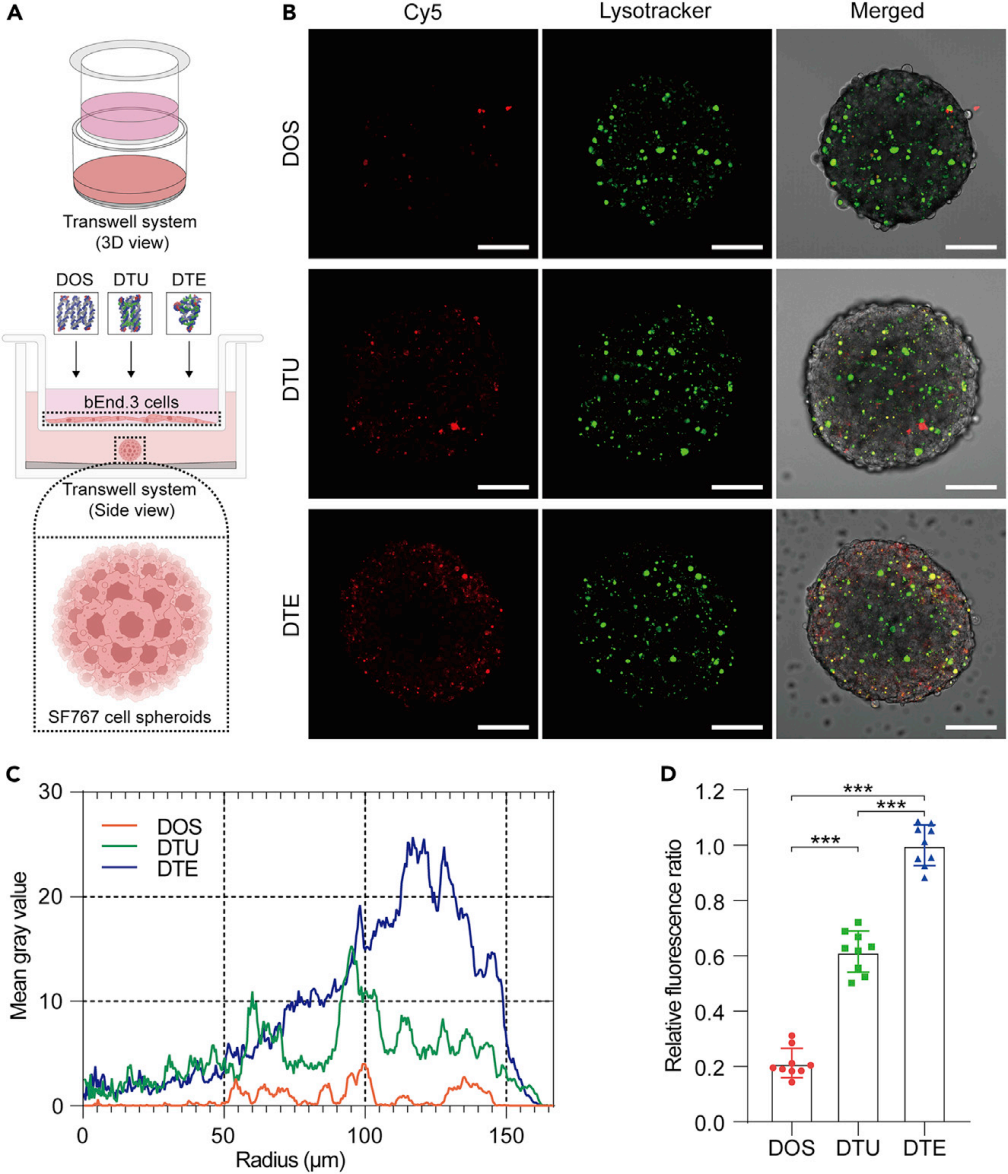
Penetration efficiency analysis of various DNA nanostructures using the *in vitro* brain tumor model (A) Schematic diagrams of the *in vitro* brain tumor model utilizing a transwell system to assess the penetration capability of DOS, DTU, and DTE. Top, the 3D view of the transwell system. The middle painting depicts different DNA nanostructures being incubated with the *in vitro* brain tumor model composed of the mono-layered bEnd.3 endothelial cells and SF767 glioma cell spheroids. The bottom illustration presents the detailed construction of 3D tumor cell spheroids. (B) Cy5-modified DNA nanostructures penetrated the SF767 tumor spheroids after crossing the BBB imitated by bEnd.3 cells. In all groups, the DNA nanostructures were treated for 6 h in the upper chambers of the transwell system we modified. Scale bars: 100 μm. (C) The fluorescence intensity of different DNA nanostructures entering the SF767 tumor spheroids was measured and compared by the Radial Profile tool of ImageJ. (D) Fluorescence statistic of the relative Cy5 intensity of DOS, DTU, and DTE in the corresponding SF767 tumor cell spheroids. The DTE group was considered the control group. The data shown represent mean ± SD from nine independent experiments. Comparisons were performed using one-way ANOVA. ∗∗∗p < 0.001.

Based on the bEnd.3 cell monolayers, we also cultivated the SF767 glioma cell spheroids in the lower dish of the transwell unit to imitate the brain tumor model *in vitro* (Figure 5A). Equimolar concentrations (10 nM) of DOS, DTU, and DTE were separately loaded into the upper chambers of brain tumor models and treated for 6 h at 37°C. After that, all treated SF767 cell spheroids were gently collected and dyed with the lysotracker for 30 min at 37°C. Confocal scanning results indicated that vast DTU and DTE permeated into the cytoplasm (Figure 5B). We then conducted radial direction fluorescence analysis on the treated cell spheroids to compare the penetrated capability of multiple DNA nanostructures in detail. The blue zone was much bigger than the orange and green zones (Figure 5C and Data S1), suggesting that DTE possessed a more robust capability to permeate cell spheroids than DTU and DOS. In addition, we respectively measured the relative fluorescence intensity of all collected cell spheroids under the BBB protection to evaluate their penetration efficiency quantitively. It is worth noting that all relative fluorescence ratio data were normalized to the average value of the DTE group. The mean fluorescence signals showed that the DOS and DTU groups were close to one-fifth and three-fifths that of the DTE group, respectively (Figure 5D), indicating the tubular and the tetrahedral variant could markedly enhance the penetration efficiency of DOS. We then integrated the penetration differences of various DNA nanostructures when permeating the SF767 cell spheroids with the protection of bEnd.3 cell monolayers (Figure 5D) or without (Figure 4D). We found that although the bEnd.3 cell monolayers prevented partial transmembrane movement of all three DNA nanostructures, the two 3D variants still improved the infiltration efficiency of DOS, especially the tetrahedral variant.

## Discussion

Most DNA nanostructures (DOS, DTU, and DTE) that entered into cells were trapped in cellular lysosomes (Figure 3A), which may be due to the short incubation time. From another perspective, it did compromise the potential of DNA nanostructures as delivery vehicles. Previous studies^32^^,^^33^ have reported that DNA structures equipped with specific nucleotide sequences and sensing probes can serve as pH reporters for dynamic acid environments in lysosomes. For existing DNA-based pH reporters, nevertheless, poor structural stability hinders the long-term monitoring of pH dynamics. Considering the high structural stability and robust transmembrane capability of DTU and DTE, these features allow them to monitor the pH dynamics when cooperating with specific probes or nucleotide sequences.

Many potential factors are shown to be able to influence the permeation efficiency of DNA-based nanostructures, such as size, configuration, molecular weight, and framework design. Among these factors, the configuration changes of DNA nanostructures inevitably impact their hydrodynamic size. Therefore, while keeping the molecular weight and lattice design of DNA nanostructures unchanged, we constructed three DNA nanostructures, including DOS, DTU, and DTE, to investigate in depth how conformational differences in DNA nanostructures affect their transmembrane capabilities. Herein, gel electrophoresis data verified that the structural stability of DTU and DTE was stronger than that of DOS. We also found that the penetration efficiency of DTU and DTE was higher than that of DOS when crossing the *in vitro* BBB and penetrating tumor cell spheroids. We hence inferred that stronger structural stability facilitates higher penetration efficiency. In particular, the tetrahedral DTE exhibited higher penetration efficiency than DTU. Considering that both “like-charge attraction”^34^ and “corner-angle-mediated molecular interaction”^13^ suggested that tetrahedral configuration had a more robust capacity to enter cells than other configurations, we should use more powerful molecular simulation models in subsequent studies to explain why DTE possesses higher transmembrane efficiency than DTU and DOS.

In summary, we proposed a strategy to enhance the transmembrane capability of DOS via folding DOS into the 3D variants, including the tubular DTU and the tetrahedral DTE. Elastic network of restraints-guided molecular dynamics simulation was employed to confirm the design feasibility and structural stability of three DNA nanostructures. DTU and DTE showed higher structural stability than planar DOS. We also systematically analyzed the transmembrane capabilities of DOS and its two variants across cerebrovascular endothelial cell monolayers into 3D brain tumor spheroids *in vitro*. The fluorescence signals of the brain tumor models treated by the three DNA nanostructures showed that the tubular and the tetrahedral configurational changes could dramatically increase the cellular uptake efficiency of the original DOS by about three and five times, respectively. It demonstrated that the 3D configurational changes could ultimately enhance the penetration capability of DOS. Thus, we believe that folding DOS into the DNA nanostructures with 3D configurations could significantly enhance its transmembrane capability. Moreover, the high structural stability, biocompatibility, penetration capability, and the enclosed inside space make the developed 3D DNA nanostructures an ideal drug delivery platform in cancer therapy, especially in crossing the BBB and permeating the brain tumors.

### Limitations of the study

Accurate AFM images are able to directly demonstrate the structural details of the designed DNA nanostructures. Other than the height features, no additional information could be observed from the existing AFM images to confirm the successful synthesis of the 3D configurations (Figure 2C). To obtain clearer AFM images, the non-contact mode in liquid rather than the peak force tapping mode in air should be considered in subsequent AFM scanning experiments.

In the future, a more comprehensive assessment of the bio-distribution and pharmacokinetics of DOS and its two variants should be performed *in vivo*. In addition, we will try to load the cellular ligands onto the developed 3D DNA nanostructures to improve their recognition capability and permeation efficiency further. We will also evaluate the efficacy of the proposed 3D DTU and DTE encapsulating the small-molecule drugs in inhibiting tumor growth. We believe that these research works can demonstrate the substantial potential of the proposed 3D DNA nanostructures assembled from DOS for target identification, transmembrane delivery, and tumor suppression.

## STAR★Methods

### Key resources table

**Table:** 

REAGENT or RESOURCE	SOURCE	IDENTIFIER
**Chemicals, peptides, and recombinant proteins**		

CellMask™ Green	Invitrogen	C37608
LysoTracker™ Green DND-26	Invitrogen	L7526
SYBR Green qPCR Mix (Low Rox)	Gene-Protein Link	G02C04S

**Critical commercial assays**		

Quick-DNA^TM^ Miniprep Plus Kit	Zymo Research	D4068

**Experimental models: Cell lines**		

Human: Hela cells	ATCC	RRID:CVCL_0030
Mouse: bEnd.3 cells	ATCC	RRID:CVCL_0170
Human: SF767 cells	ATCC	RRID:CVCL_6950

**Oligonucleotides**		

Single-stranded scaffold DNA	This paper	N/A
Multifunctional staple strands DNA	This paper	N/A
Primers for qPCR	This paper	N/A

**Software and algorithms**		

caDNAno	Douglas et al., 2009^22^	https://cadnano.org
Mrdna framework	Maffeo and Aksimentiev, 2020^24^	https://gitlab.engr.illinois.edu/tbgl/tutorials/multi-resolution-dna-nanostructures
NAMD	Phillips et al., 2005^35^	https://www.ks.uiuc.edu/Research/namd/
VMD	Humphrey et al., 1996^36^	https://www.ks.uiuc.edu/Research/vmd/
ImageJ	Schneider et al., 2012^37^	https://imagej.nih.gov/ij/
GraphPad Prism 8	GraphPad software	https://www.graphpad.com/scientific-software/prism/

### Resource availability

#### Lead contact

Further information and requests for resources should be directed to and will be fulfilled by the corresponding author: Xiaoming Liu (liuxiaoming555@bit.edu.cn).

#### Materials availability

This study did not generate any new reagents.

### Experimental model and subject details

#### Cell lines

The Hela cervical cancer cells, the bEnd.3 cerebral endothelial cells, and SF767 brain tumor cells were purchased from American Type Culture Collection (ATCC, USA). bEnd.3 cells were cultured with RPMI 1640. Hela cells and SF767 cells were cultivated in Dulbecco’s modified Eagle’s medium (DMEM). All cell culture media were supplemented with 10% fetal bovine serum (Gibco, USA), 100 U/mL penicillin, 100 μg/mL streptomycin, and 1 × GlutaMAX (Gibco, USA). All cells were maintained in a humidified incubator at 37°C with 5% CO_2_.

### Method details

#### Molecular simulations

Using caDNAno,^22^ we designed the following three DNA origami nanostructures (Figure S1): a flat DOS with a size of 14 nm × 15 nm × 2 nm; a DNA tube (DTU) bent by the flat DOS through the red folding axis (Figure S2); and a DNA tetrahedron (DTE) folded by DOS through green folding axes (Figure S2). These DNA designs were firstly predicted using the mrdna framework proposed by Aksimentiev and colleagues.^24^ The original caDNAno documents were imported into the framework, respectively. We employed the multiresolution simulations method as previously described.^21^ The whole process is divided into three stages. At first, 4 base pair (bp)/bead models of these objects were created and used in 20 μs simulations to reveal the fluctuations of the structures, assuming the self-assembly and folding direction occurred as designed. The structural configurations at the end of the simulations were used to update splines representing the centerline of each helix. Secondly, the higher resolution 2 beads/bp models with an explicit, local representation of the twist in each helix were generated and relaxed during 400 ns simulations. In the third stage, the conformations from the last 5 ns of the simulations were averaged and used to update the configuration of the beads for the final simulations with the linking number of each helix held fixed by harmonic twist dihedral angle potentials. The NAMD^35^ conformations of our three DNA nanostructures at the end of the final mrdna stage were performed by the Elastic Network of Restraints Guided molecular dynamics simulations.^25^ In the study, base pairs were considered intact when the bases fell within a 5 Å cutoff. All simulated animations were monitored by VMD.^36^

#### Preparation of DNA origami nanostructures

All DNA oligonucleotides were purchased from Sangon Biotech (Shanghai, China). Considering the synthesis limitation (max length: 150 nt) of oligonucleotides, we carefully divided the whole scaffold DNA into two parts: Scaffold 1 (126 nt) and Scaffold 2 (126 nt) to decrease the breakpoint number of whole scaffold DNA (252 nt) as far as possible (Table S1). Two single-stranded scaffold DNA (Table S1) were mixed with common staple strands (for DOS, DTU, and DTE), folding strands (for DTU and DTE) and Cy5 capture strands (for DOS, DTU, and DTE) in the reaction buffer (10 mM Tris, 1 mM EDTA, and 10 mM MgCl_2_) at a molar ratio of 1:10, respectively (Table S2). These mixtures were annealed in the thermal cycler (Bioer, China) at 95°C for 30 s, followed by a temperature decrease of 10 min/°C until 65°C, and afterward a decrease of 20 min/°C until 20°C. After the annealing, we then chose the Amicon Ultra-0.5 mL 30 KD centrifugal filters (Millipore Corporation, USA) to filtrate the above solutions three times at 5000 rpm for 3 min. To observe these DNA nanostructures in the following fluorescent experiments, we incubated them with Cy5 strands (5′-TAAACTCTTTGCGCAC-Cy5-3′) at a molar ratio of 1:5. These mixtures were heated to 45°C, then cooled to 20°C at a steady rate of 10 min/°C. Excess Cy5 strands were removed from the above mixture through filtration using the Amicon Ultra-0.5 mL 30 KD centrifugal filters. The ultrafiltration (5000 rpm, 3 min) for each DNA sample was performed at least three times.

#### Atomic force microscopy

All DNA nanostructures were characterized using atomic force microscopy (AFM). Briefly, DNA samples (10 nM, 5 μL) were adsorbed on freshly cut micas for 5 min. Then, the excessed solutions were absorbed by filter papers. The micas were then washed with distilled deionized water three times and dried in a nitrogen atmosphere. Finally, the images of the above micas covered with DNA samples were captured by using a Bruker Dimension® Icon^TM^ instrument (Bruker, Germany) and then analyzed with the NanoScope-Analysis software.

#### Structural stability assay

In the physiological environment: The working solution for each DNA nanostructure was adjusted to 20 nM and then mixed with DMEM (supplemented with 20% FBS) at a 1:1 volume ratio. All samples were immediately incubated at 37°C for 2, 4, and 6 h.

In the low-pH environment: To mimic the cleavage situation of DNA nanostructures in cellular lysosomes, DOS, DTU, and DTE were treated in the low-pH environment (pH = 5) for different periods at 37°C.

Right after incubation, different DNA samples were subjected to 10% native polyacrylamide gel electrophoresis (PAGE) at 100 V for 1 h (gel prepared in 1 × TBE buffer supplemented with 10 mM MgCl_2_). After the electrophoresis, the gels were carefully recovered and incubated with 1 h in the staining solution (3 × SYBR Safe was dissolved in 1 × TBE buffer). Finally, the stained gels were exposed using the Tanon 4600SF multifunctional imaging system (Tanon, China).

#### Confocal microscopy

Hela cells were seeded on confocal culture dishes at a 5 × 10^5^ cells/ml density and grown overnight. The cells were gently washed twice with phosphate buffer (PBS) and incubated with free Cy5-strands in a fresh cell culture medium for 6 h at 37°C. After cultivation, cells were carefully rinsed twice with PBS. Cell membrane and cell lysosome were stained by CellMask Green (Invitrogen, USA) and LysoTracker Green DND-26 (Invitrogen, USA) for 30 min, respectively. Then, treated cells were mildly washed with PBS three times, and 1 mL fresh cell culture medium was added before the confocal images were captured using the confocal microscope (Nikon C2, Japan). Finally, all confocal images were analyzed by ImageJ.^37^

#### Flow cytometry

1 × 10^6^ Hela cells were inoculated in each well of a 6-well plate and grown overnight. Then, the cells were washed twice with PBS and treated with Cy5-labeled DOS, DTU, and DTE for 2 h, 4 h, and 6 h at 37°C. Additional Hela cells treated with the free Cy5-strands at different time intervals were considered the control group. After that, the above cells were harvested and rinsed three times with PBS. All treated cells were kept in fresh PBS and sufficiently resuspended before the fluorescence intensity was determined by flow cytometry (BD biosciences, USA).

#### DNA extraction and qPCR analysis

Before treating Hela cells with different DNA nanostructures, well-grown Hela cells were inoculated in 6-well plates. Approximate 1.0 × 10^6^ cells in every well were rinsed by PBS three times and incubated with equal-molar (10 nM) DOS, DTU, and DTE respectively. After 2 h of incubation, DNA nanostructures-treated Hela cells were rinsed with PBS three times and then collected separately. Total DNA was isolated with the Quick-DNA^TM^ Miniprep Plus Kit (Zymo Research, USA). 1 μg of total DNA was mixed with 2 × SYBR Green qPCR Mix (Low Rox). The following quantitative PCRs were conducted on the QuantStudio^TM^ 3 instrument (Applied Biosystems, USA). The β-actin (a housekeeping gene) was chosen as the reference gene. Considering that the “target gene” of all three DNA nanostructure-treated cell groups should maintain consistency, the scaffold 2 (Table S1) was set as the “target gene”. The target gene (Scaffold 2) levels were normalized to β-actin. All used primers were listed as follows.

β-actin forward: 5′-GCTCGTCGTCGACAACGGCT-3’
β-actin reverse: 5′-CAAACATGATCTGGCTCATCTTCTC-3’
Target gene (Scaffold 2) forward: 5′- TATATCGGTTATGCGTGGG-3’
Target gene (Scaffold 2) reverse: 5′-CGGTTTATCAGCTTGCTTT-3′

#### Preparation of cell spheroids

The three-dimensional (3D) SF767 cell spheroids were fabricated, as described in the previous study.^38^ Briefly, before each 3000 cells were seeded into each well of the 96-well plate, each well had been preloaded with 100 μL of 1.5% sterile agarose solution. The cell culture medium was replaced every two days until the diameter of cell spheroids became 300 μm approximately. The formation of 3D cell spheroids was monitored using a universal optical microscope.

#### Construction of BBB model *in vitro*

The *in vitro* BBB monolayer was constructed with bEnd.3 cells based on the previous report.^17^ Briefly, bEnd.3 cells (1 × 10^4^ cells/well) were inoculated onto the gelatin solution-treated upper chamber of a transwell system and cultured with RPMI 1640 containing 10% FBS for one week. The integrity of cell monolayers was measured with a Millicell-ERS voltohmmeter (Millipore, USA). When the transendothelial electrical resistance value of the monolayer exceeded 300 Ω cm^2^, the upper chamber was considered as an *in vitro* BBB model for further experiments.

#### Penetration efficiency assay

For cell spheroids, 300 μL of 1.5% sterile agarose solution was added to each well (24-well plate) to prevent the cell spheroids from adhering to the substrate. After that, the SF767 cell spheroids were individually placed into the cell culture plates. Cy5-modified DNA nanostructures were incubated separately with cell spheroids for 6 h at 37°C. All cell spheroids were rinsed three times with PBS buffer prior to stereo scanning using confocal microscopy.

For cell spheroids under BBB protection: Based on the *in vitro* BBB model we modified, 300 μL of 1.5% sterile agarose solution was loaded in each low chamber. After cooling at room temperature for 30 min, the 3D cell spheroids were gently relocated into the low chamber. To evaluate the transmembrane efficiency of DOS, DTU, and DTE across BBB *in vitro*, three Cy5-modified DNA nanostructures (10 nM) were separately added into the upper chambers of constructed before. After 6 h of incubation, the upper chambers were removed. Next, the 3D SF767 cell spheroids were carefully washed three times with PBS. The fluorescence intensity of all cell spheroids was observed using the confocal microscope (Nikon C2, Japan).

### Quantification and statistical analysis

There were at least three independent experiments in each statistical analysis. Results are presented as mean ± SD. One-way ANOVA was used to evaluate the statistical significance of the differences among the different groups. When ANOVA was significant, a *P*-value less than 0.05 is considered statistically significant.

## Data Availability

Data reported in this paper will be shared by the lead contact upon request. This study does not report any original code. Any additional information required to reanalyze the data reported in this paper is available from the lead contact upon request.

## Supplemental information

Document S1. Figures S1–S7 and Tables S1 and S2

Data S1. Raw data in the main text, related to Figures 2, 3, 4, and 5
