# Supplementary material for: Transmembrane capability of DNA origami sheet enhanced by 3D configurational changes
Source: iScience. 2023 Feb 15;26(3):106208. doi: 10.1016/j.isci.2023.106208 (PMC9982283; doi:10.1016/j.isci.2023.106208)
Supplement: Document S1. Figures S1–S7 and Tables S1 and S2 [file mmc1.pdf]

iScience, Volume *26*

Supplemental information

Transmembrane capability of DNA origami sheet enhanced by 3D configurational changes

Fengyu Liu, Xiaoming Liu, Wendi Gao, Libo Zhao, Qiang Huang, and Tatsuo Arai

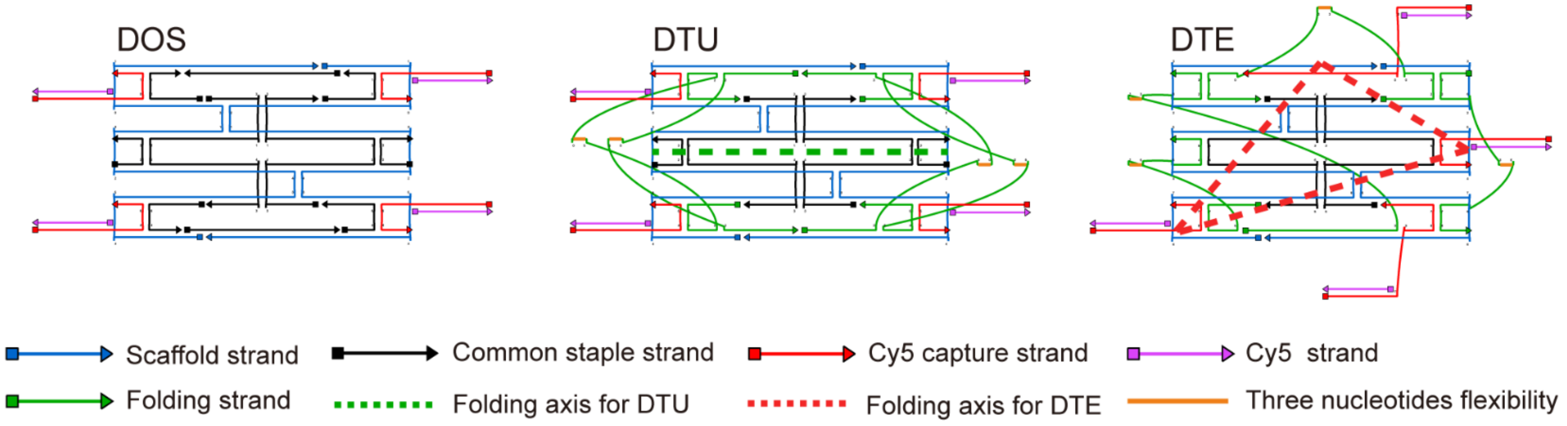

Figure S1. Design schemes of three DNA nanostructures in caDNAno, related to Figure 1. The two scaffold strands (blue) and common staple strands (black) and Cy5 capture strands (red) were mixed and annealed to assemble DOS. DTU and DTE were constructed by bending DOS through particular folding strands (green). Three nucleotides (orange) were introduced into the middle fragment of all folding strands to relieve the helix stresses caused by folding procedures. Alternatively, Cy5 strands (purple) were conjugated to DOS, DTU, and DTE by the hybridization between Cy5 capture strands and Cy5 strands. The potential folding axes formed by the folding procedures of DTU and DTE were labeled by green and red dashed lines, respectively.

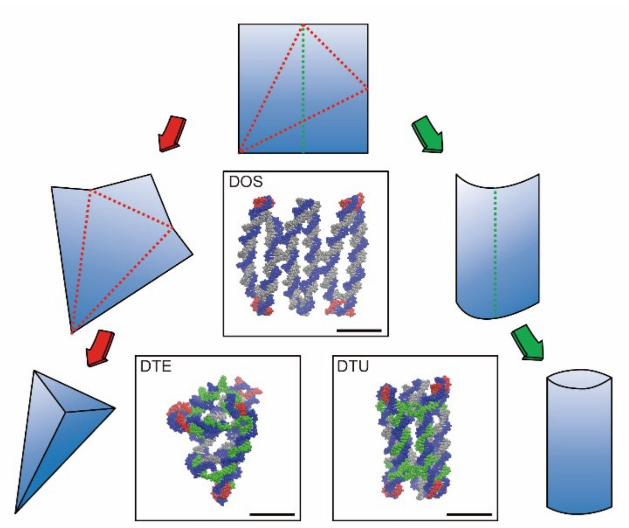

Figure S2. Construction pathways of DTU and DTE utilizing the DOS template, related to Figure 1. When the folding strands of DTU were added into the synthesized process (green pathway), the whole DOS structure would fold into the tubular configuration along with the green dotted folding axis. Similarly, the folding strands of DTE were annealed with traditional staple strands and further bent into the tetrahedral geometry according to the red-dotted folding axes (red pathway). The representative simulated images demonstrated the rectangular, tubular, and tetrahedral topology of DOS, DTU, and DTE.

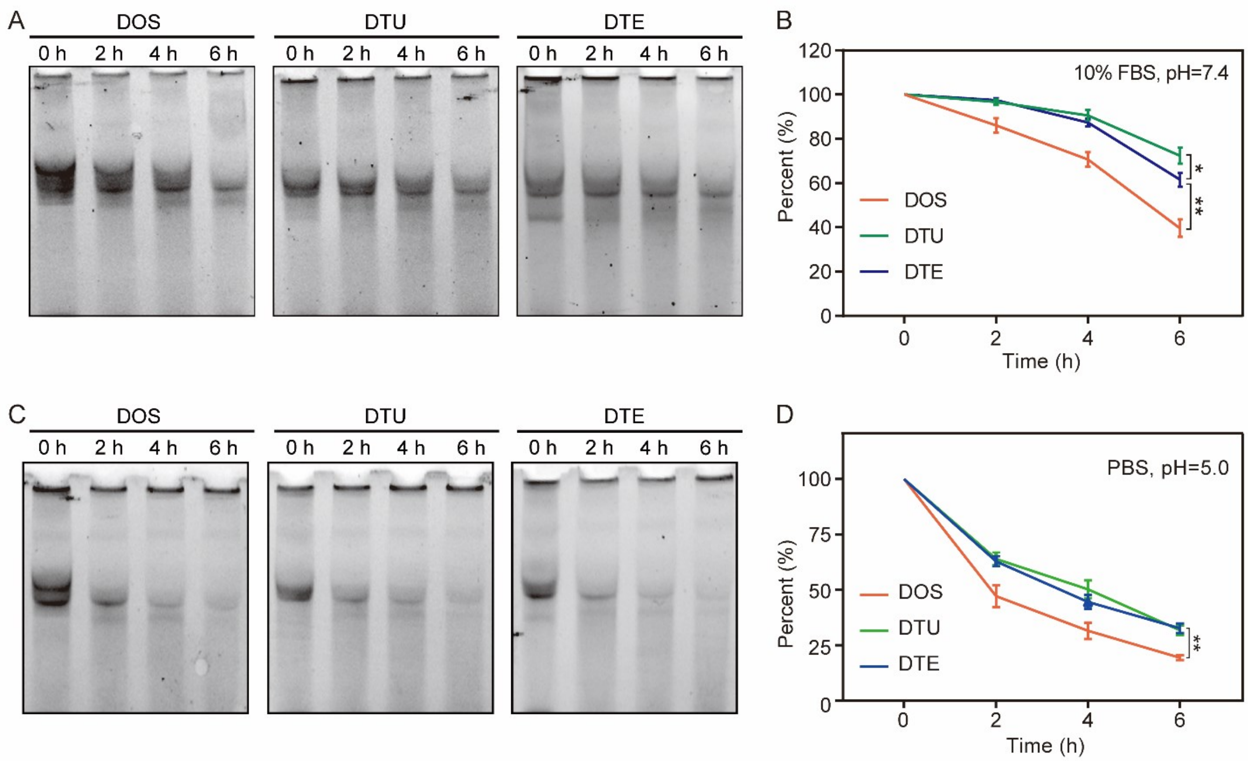

Figure S3. Representative gel electrophoresis images of three DNA nanostructures treated with 10% FBS (A) and low pH solution (C) at different time courses (0, 2, 4, and 6 h), related to STAR Methods. The loading volume of each lane was 20 μL. (B, D) The statistical analysis results showed that three DNA nanostructures exhibited various structural stability when incubated in different environments. All detected signals of treated samples (2 h, 4 h, and 6 h) were normalized to the detected signals of control samples (0 h). The data shown represent mean ± SD from three independent experiments. Comparisons were made using one-way ANOVA. *$p < 0.05$, **$p < 0.01$.

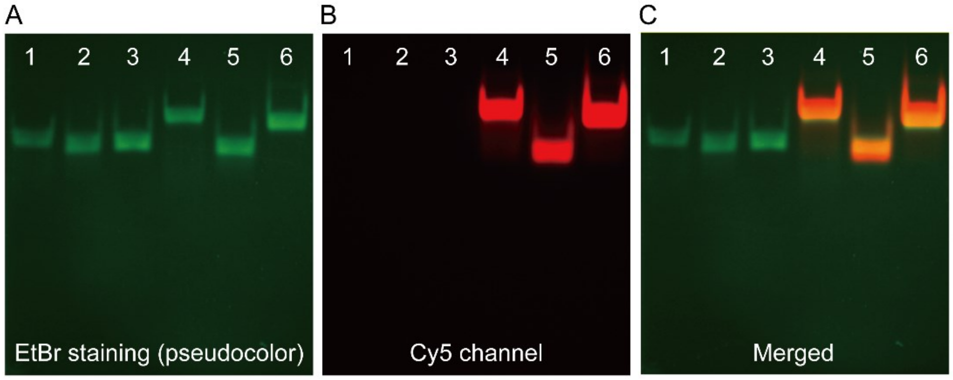

Figure S4. Co-localization of Cy5 dyes and diverse DNA nanostructures, related to Figure 2. Lane 1: DOS; Lane 2: DTU; Lane 3: DTE; Lane 4: Cy5-DOS; Lane 5: Cy5-DTU; Lane 6: Cy5-DTE. Compared with the tetrahedral shape of DTE and the planar structure of DOS, the tubular configuration of DTU is beneficial to move faster in the polyacrylamide gel. Therefore, the partial retardation of Cy5-DTU caused by the attachment of Cy5 strands has been neutralized. Diverse DNA nanostructures were incubated with Cy5 strands for 2 h at room temperature. All samples have been filtered three times using 30 KDa ultrafiltration devices before loading to different lanes. The electrophoresis experiment was repeated at least three times.

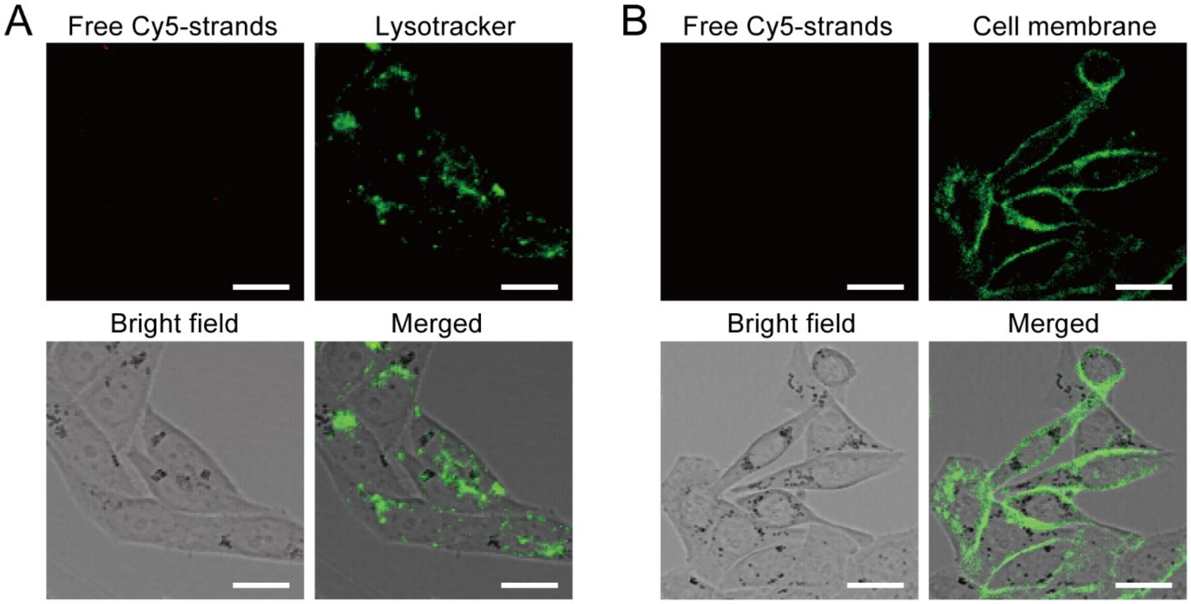

Figure S5. Representative Confocal images showed the free Cy5-strands were rarely observed in HeLa cells (A) and bEnd.3 cells (B), related to Figure 3. The cultivation time: 6 h. Scale bars: 10 μm.

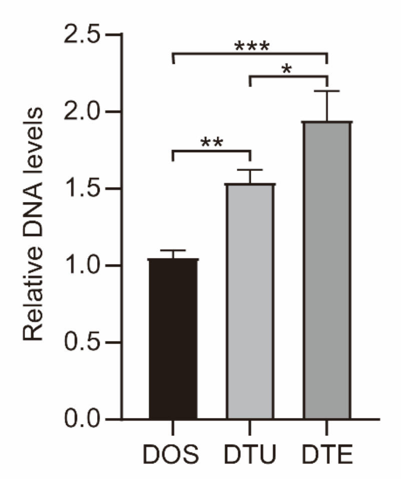

Figure S6. DTU and DTE have stronger cellular uptake efficiency than DOS, related to STAR Methods. All detected DNA levels of the target gene (Scaffold 2) from three groups were normalized to β-actin. The data shown represent mean ± SD from three independent experiments. Comparisons were made using one-way ANOVA. *$p < 0.05$, **$p < 0.01$, ***$p < 0.001$.

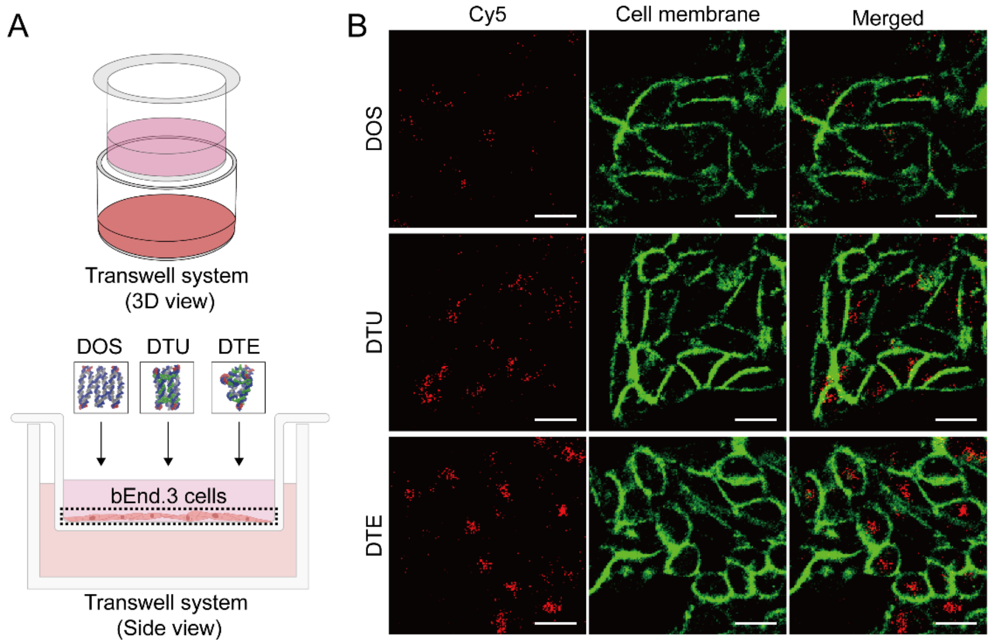

Figure S7. Localization of multiple DNA nanostructures internalized into bEnd.3 cells, related to Figure 4. (A) Schematic diagrams of the in vitro BBB model utilizing a transwell system to assess the penetration capability of DOS, DTU, and DTE. Top, the 3D view of the transwell system. The bottom painting depicts different DNA nanostructures being incubated with the mono-layered bEnd.3 endothelial cells. (B) The equimolar (10 nM) of Cy5-modified DOS, DTU, and DTE were separately incubated with the mono-layered bEnd.3 cells for 6 h. After that, all treated bEnd.3 cell membranes were stained using the CellMask working solution. These typical images were randomly selected from three independent experiments. Scale bars: 20 μm.

Table S1. Detailed sequences of two single-stranded scaffold DNA, related to STAR Methods.

Name	Sequence (5’-3’)
Scaffold 1	TAATGGAAACTTCCTCATGAAAAAGTCTTTAGTC<br>CTCAAAGCCTCTGTAGCCGTTGCTACCCTCGTT<br>CCGATGCTGTCTTTCGCTGCTGAGGGTGACGA<br>TCCCGCAAAAGCGGCCTTTAACTCCCT
Scaffold 2	GCAAGCCTCAGCGACCGAATATATCGGTTATGC<br>GTGGGCGATGGTTGTTGTCATTGTCGGCGCAA<br>CTATCGGTATCAAGCTGTTTAAGAAATTCACCT<br>CGAAAGCAAGCTGATAAACCGATACAAT

Table S2. Detailed sequences of the multifunctional staple strands of DOS, DTU, and DTE, related to STAR Methods.

Object	Strand type	Sequence (5'-3')
DOS	Common staple strands	GGCCGATCGTCAC
		TTATCCGAGGTGA
		CATCGGACAG
		CCTCAACCGACAACAGCAGCGAAAGAACGAGGGTAGCA ACGCTTTGAG
		ATTCAGAGGGCTATCTTAAACAGCTAGTTGCGCCGACAA TATCGCCCA
		GACTAAAGGAGGA
		ACCGATTGAT
		AGTTTCCATTAATTGTATCGGT
		CGCATAACGGTCG
		CTGAGGCTTGCAGGGAGTTAAA
	Cy5 capture strands	GTGCGCAAAGAGTTTATTTTCGGGCGATA
		GTGCGCAAAGAGTTTAATAGCCTTATTAC
		GTGCGCAAAGAGTTTATTCGATTTCGTGG
		GTGCGCAAAGAGTTTATTTCATACTTACA
DTU	Common staple strands	CATCGGACAG
		ACCGATTGAT
		ATTCAGAGGGCTATCTTAAACAGCTAGTTGCGCCGACAA TATCGCCCA
		CCTCAACCGACAACAGCAGCGAAAGAACGAGGGTAGCA ACGCTTTGAG
	Folding strands	AGGGAGTTAAATTTAGTTTCCATTA
		CGCATAACGGTCGATATTATCCGAGGTGA
		ATTGTATCGGTTATCTGAGGCTTGC
		GACTAAAGGAGGAAATGGCCGATCGTCAC
	Cy5 capture strands	GTGCGCAAAGAGTTTATTCGATTTCGTGG
		GTGCGCAAAGAGTTTAATAGCCTTATTAC
		GTGCGCAAAGAGTTTATTTTCGGGCGATA
		GTGCGCAAAGAGTTTATTTCATACTTACA
DTE	Common staple strands	CCTCAACCGACAACAGCAGCGAAAGAACGAGGGTAGCA ACGCTTTGAG
		ATTCAGAGGGCTATCTTAAACAGCTAGTTGCGCCGACAA TATCGCCCA
	Folding strands	GACTAAAGGAGGATATCATCGGACAG
		CGCATAACGGTCGAAAGGCCGATCGTCAC
		TATTCCGATATTAGCTTTAGCTT
		AGTTTCCATTAATTGTATCGGTATAGCGGGCTTTT
	Cy5 capture strands	GTGCGCAAAGAGTTTATTTCATACTTACA
		GTGCGCAAAGAGTTTAAGTGGAGCCTATT
		GTGCGCAAAGAGTTTATAGTTAGCCAGCT
		GTGCGCAAAGAGTTTAAAATTGAGGGACGTTCGGAGTC
Cy5 strand		TAAACTCTTTGCGCAC-Cy5
